# Thrombosis of the Azygos Anterior Cerebral Artery

**DOI:** 10.1155/2017/5409430

**Published:** 2017-02-12

**Authors:** Camila Soares Moreira de Sousa, Carla Lorena Vasques Mendes de Miranda, Marcelo Coelho Avelino, Breno Braga Bastos, Rafael Soares Moreira de Sousa, Carla Valéria Vasques Mendes de Miranda

**Affiliations:** ^1^Division of Radiology, Medimagem, Teresina, PI, Brazil; ^2^Division of Radiology, Emergency Hospital in Teresina Dr. Zenon Rocha, Teresina, PI, Brazil; ^3^Division of Radiology, UDI 24 Hours, Teresina, PI, Brazil; ^4^Division of Radiology, Antonio Prudente Hospital, Fortaleza, CE, Brazil; ^5^Division of Radiology, UDI Hospital, São Luís, MA, Brazil

## Abstract

The azygos anterior cerebral artery is a rare variant, characterized by the absence of the anterior communicating artery and the union of two proximal segments of the anterior cerebral artery, forming a single trunk and ascending through the interhemispheric fissure. The incidence in the population varies from 0.3 to 2%. The presence of occlusion for this vessel causes bifrontal infarcts, with potentially devastating functional consequences, hence the importance of recognizing this anatomical variation in imaging exams.

## 1. Introduction

The azygos anterior cerebral artery (AACA) is a rare anomaly of the circle of Willis with an incidence of 0.3–2% in the overall population. It is characterized by a single vessel which provides the blood supply to both anterior cerebral hemispheres. This anatomical variation has a significant impact on blood hemodynamics of the frontal lobe, especially in cases of occlusion, determining bifrontal infarction [1–3].

## 2. Case Report

**Figure 1:**
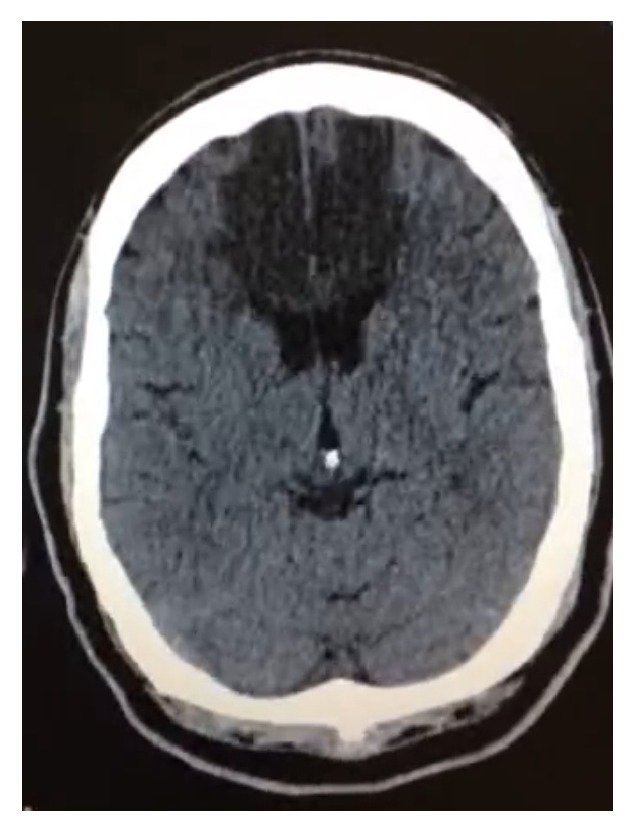
Cerebral computerized tomography scan, axial view, showing image of ischemic cerebral stroke in typical irrigation territory of the bilateral anterior cerebral artery.

**Figure 2:**
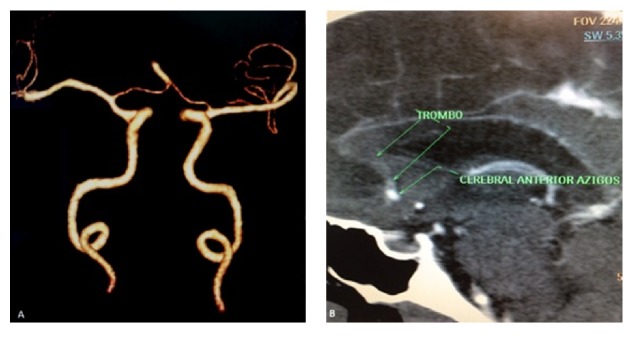
3D reconstruction of cerebral angiotomography (A) and sagittal reformatting (B) showed azygos anterior cerebral artery with endoluminal thrombus, about 1 cm of its common origin, reducing the vascular flow completely.

Male patient, 63 years old, previously hypertensive and diabetic, admitted to the emergency room with a sudden lowering of the level of consciousness. He went through cerebral computerized tomography that showed ischemic cerebral stroke image in the typical irrigation territory of the bilateral anterior cerebral artery (ACA), with characterization of a single hyperdense vessel for a probable topography of ACA (Figure 1). Cerebral angiography showed AACA with endoluminal thrombus, about 1 cm of its common origin, reducing the vascular flow completely, determining an ischemic stroke in the frontal lobe bilaterally and anterior regions of the corpus callosum (Figure 2). The patient presented worsening from the clinical point of view, evolving to death during hospitalization.

## 3. Discussion

The cerebral arterial circle, also known as the circle of Willis, is a complex anastomotic network between the internal carotid artery and vertebrobasilar systems; it is located in the skull base and is home to numerous anatomical variations. The anterior circulation consists of internal carotid arteries, which originate from the carotid bifurcation and are divided into anterior cerebral and middle cerebral arteries. The anterior cerebral arteries interconnect to the anterior communicating artery whose patency allows interhemispheric potential shunts in case of obstructions or stenoses in the vascular network [4].

The AACA is a rare variant, characterized by the absence of the anterior communicating artery and the union of the two proximal segments (A1) of the anterior cerebral arteries, forming a single stem (A2) and ascending through the interhemispheric fissure [1–3]. The occurrence of occlusion of this vessel determines bifrontal infarctions, with potentially devastating functional consequences, so it is important to recognize this anatomical variation in imaging exams.

In 1963, Baptista systematized the most frequent anomalies of the distal segments of the anterior cerebral arteries and showed an incidence of 0.3% AACA among the brains he studied [5].

The anatomical recognition of this variant becomes important for the occurrence of saccular aneurysms, relatively common (13–71%), associated malformations (dysgenesis of the corpus callosum, lobar holoprosencephaly, septooptic dysplasia, porencephalic cysts, and arteriovenous malformations), and presence of bifrontal infarctions in case of occlusion [1].

The actual prevalence of anterior circulation anomalies among the general population is still unknown; however, it becomes extremely important to recognize their anatomy and variations, playing an important role in the planning of neurosurgical procedures, reducing iatrogeneses and their complications.
